# GASTRODIET 2023, an international meeting on food, diet and gastrointestinal health, held 18–20 October 2023 at Monash University Prato Centre, Prato, Italy

**DOI:** 10.1002/jgh3.13106

**Published:** 2024-07-01

**Authors:** Jane G Muir

**Affiliations:** ^1^ Department of Gastroenterology, School of Translational Medicine Monash University Melbourne Victoria Australia

After successful hosting two GASTRODIET conferences in 2015 and 2017, and following a six‐year hiatus, the GASTRODIET 2023 meeting was finally organized again from the 18th to the 20th of October 2023 at the beautiful Monash University Prato Centre. The Monash University Prato Centre is in the heart of the small medieval town of Prato, nestled in Tuscany, and only 20 min by train from Florence. Located in the 18th – century palace, the *Palazzo Vaj*, the Monash University Prato Centre provided the perfect venue for the 117 conference attendees. As the name suggests, this meeting is all about gastroenterology and diet. It brought together dietitians, gastroenterologist, and scientists from 28 countries across Asia, Europe, North and South America, Australia, and New Zealand.

The keynote address and JGH Foundation Lecture were given by Professor Eamonn Quigley (*Weill Cornell Medical College at Houston Methodist Hospital*, *USA*), which addressed the question ‘Can Diet Change the Natural History of Gastrointestinal Diseases?’ It provided the perfect insight and perspective and set the tone for the conference.

Since our last GASTRODIET meeting in 2017, there are a number of areas that are clearly advancing (evolution) and in some areas major innovation (revolution) is taking place.

The global adoption of the low FODMAP diet as a first‐line treatment for IBS highlights its evolution. This evolution is also evident in the ‘fine‐tuning’ of the diet to optimize its benefits, minimize potential risks, and enhance overall health parameters. Emphasizing the significance of the three phases of the diet program and introducing the concept of the less restrictive or ‘FODMAP gentle’ and the Mediterranean‐style low FODMAP version of the diet have all been part of the evolution. Additionally, the diet's evolution has included the acknowledgement that IBS‐like symptoms overlap with other conditions, leading to the testing of the diet in non‐IBS populations such as the elderly and women with endometriosis as well as functional dyspepsia. Evolution in FODMAP diet therapy is also evident through understanding how manipulation of dietary fibers and the use of oral enzyme supplementation can be employed as adjunct therapies.

The diagnosis and management of coeliac disease are also evolving, with novel immune biomarkers being identified that may be used for diagnosis and avoid the need for the oral gluten challenge.

In relation to the role of diet in IBD (Crohn's disease and ulcerative colitis), we find ourselves in the ‘grip of a revolution.’ Evidence is emerging regarding the pivotal role diet may play as a significant environmental factor in IBD susceptibility, as well as in the course of established IBD. As we ‘watch this space,’ we eagerly await the outcome of several studies currently underway.

The ongoing evolution, and indeed revolution, of diet therapies in the management of gastrointestinal disorders necessitates new approaches to ‘measuring’ and probing the workings of the gut. Indeed, advances are being made. Innovative techniques under development include telemetric ingestible devices such as the gas‐sensing capsule, analysis of fecal‐derived volatile organic compounds (fecal ‘sniffing’), luminal ‘tasting’ and the application of intestinal ultrasound.

One of the key takeaways from the GASTRODIET conference emphasizes the important role of the dietitian, as highlighted in the final debate, always a highlight of these gatherings, between Professor Peter Gibson (*Monash University, Australia*) and Professor Kevin Whelan (*Kings College London, United Kingdom*) titled ‘A Dietitian Is Essential in Delivering a Therapeutic Diet.’ The clear victory for the affirmative (supporting the importance of dietitians) clearly reinforces the pivotal role of dietetics and dietitians, solidifying the conference's overarching message regarding the involvement of diet therapy and multidisciplinary teams. This victory underscores the importance of dietitians working collaboratively within multidisciplinary teams to effectively manage their patients.

Audience engagement and participation at the GASTRODIET meetings is always a feature of these conferences. The small size of the meeting, relaxed nature of the venue, and friendly atmosphere always ensure excellent audience participation, with questions following each session. We would like to express our gratitude to our sponsors: the JGH Foundation, Modifyhealth, Atmo Biosciences, Dr. Schär, and the NHMRC Centre of Research Excellence in Digestive Health. Additionally, we extend our appreciation to the Monash Prato Event organizing team led by Sarah Gore, and the wonderful food catering, which is always a highlight of this event, with the Mediterranean diet well represented. We thank all the speakers, moderators, event organizers, the Monash FODMAP team, and all attendees for participating in and contributing to the success of the GASTRODIET 2023 meeting.

Within this special JGH Open supplement are papers from the speakers that summarize the GASTRODIET 2023 talks. We thank JGH Foundation for sponsorship and making this possible.

We look forward to meeting again in Prato at GASTRODIET 2025.

